# A systematic review of one-lung ventilation during thoracic surgery comparing the safety and efficacy of high and low tidal volumes

**DOI:** 10.1186/1749-8090-10-S1-A108

**Published:** 2015-12-16

**Authors:** Julian Camilleri-Brennan, Nandesh Patel, Sarah Mok, David Ryan, Blair Wilson, Mikolaj Kotts, Olivia Morton, Mong Tung, Peter Alston

**Affiliations:** 1 The University of Edinburgh, College of Medicine and Veterinary Medicine, 47 Little France Crescent, Edinburgh, EH16 4TJ, UK; 2 Department of Anaesthesia, Critical Care and Pain Medicine, Royal Infirmary of Edinburgh, 51 Little France Crescent, Edinburgh, EH16 4SA, UK

## Background/Introduction

Lung isolation, a technique largely used to facilitate access during thoracic surgery, can create some difficulty in maintaining a patient's blood gas balance. Strategies have been used to overcome the ventilation-perfusion mismatch associated with one-lung ventilation (OLV). However, such strategies may induce volutrauma, barotrauma and atelectotrauma in the ventilated lung, leading to acute lung injury (ALI) and/or acute respiratory distress syndrome (ARDS). Different ventilatory parameters have been used to improve the safety and efficacy of OLV, with the use of high or low tidal volumes (VT) being the most contentious.

## Aims/Objectives

The aim of this study was to undertake a systematic review and meta-analysis of the literature comparing the safety and efficacy of OLV using high and low VT ventilation.

## Method

A comprehensive literature search was performed on EMBASE, Web of Science and MEDLINE, from inception until October 2014 to identify studies comparing of high and low VT strategies for OLV. The systematic review of papers used the PRISMA 27-step checklist. [1]

## Results

Our search yielded twelve studies. To measure safety, five studies considered ALI/ARDS while four measured the release of pro-inflammatory cytokines. Significant association was found between a high VT and ALI/ARDS in two of these studies. Shunting and oxygenation were considered the primary measurements of efficacy in three and four of the studies found respectively. Two studies each considered shunting and oxygenation and found that lower VT were associated with more adverse effects than higher VT settings. Studies were not comparable, as they used dissimilar co-interventions so a meta-analysis could not be conducted.

## Discussion/Conclusion

To date, RCTs comparing high and low VT during OVL are limited and flawed; however, weak evidence indicates that lower may be safer than higher VT. Future research requires larger sample sizes, the standardisation of definitions of high or low VT and co-interventions before optimal strategies can be determined.
